# Multifocal Biliary Adenoma Involving the Gallbladder and Common Bile Duct Presenting With Obstructive Jaundice: A Case Report

**DOI:** 10.7759/cureus.93487

**Published:** 2025-09-29

**Authors:** Austin Guadarrama, Tyler Mouw, Edwin O Onkendi, Mohamad Sidani, Kanak Das, Luis Brandi

**Affiliations:** 1 Department of Medicine, Texas Tech University Health Sciences Center, Lubbock, USA; 2 Department of Surgery, Texas Tech University Health Sciences Center, Lubbock, USA; 3 Department of Gastroenterology, Texas Tech University Health Sciences Center, Lubbock, USA; 4 Department of Pathology, Texas Tech University Health Sciences Center, Lubbock, USA

**Keywords:** adenoma, common bile duct, obstructive jaundice, pancreaticoduodenectomy, tubulovillous

## Abstract

Biliary adenomas are rare neoplastic processes of the gallbladder and extrahepatic biliary tree that are pre-malignant. These tumors infrequently present with obstructive jaundice, which is more suggestive of underlying invasive malignancy. Here, we present a case of multifocal biliary adenoma without invasive cancer in a 70-year-old man who presented with jaundice. Initial laboratory results and imaging suggested a malignant process, prompting surgical resection. Intraoperative findings and histopathologic examination revealed a multifocal tubulovillous adenoma involving the gallbladder and distal common bile duct. The patient ultimately underwent a curative pancreatoduodenectomy procedure. This case demonstrates multifocal biliary adenoma presenting as jaundice without underlying malignancy. Surgical treatment is necessary to relieve symptoms and prevent the progression to carcinoma.

## Introduction

Biliary adenomas are rare benign neoplasms arising from the epithelial lining of the gallbladder or extrahepatic biliary tree [1, 2]. Extrahepatic biliary adenomas are particularly uncommon, accounting for only six percent of all extrahepatic bile duct masses [1]. Despite their low prevalence, these lesions are clinically significant due to their potential for malignant transformation [3, 4]. Most biliary adenomas are asymptomatic and are detected incidentally on imaging or on histopathology post-cholecystectomy [1, 2, 5]. However, a minority of patients may present with abdominal pain and obstructive jaundice if lesions grow large enough to cause significant obstruction of the biliary tract [3, 5, 6]. This poses a diagnostic challenge, as extrahepatic biliary adenomas are difficult to distinguish from other malignant pathologies causing biliary obstruction [1, 5, 7]. Thus, endoscopic evaluation and surgical excision are required for definitive diagnosis and treatment [3, 5, 8]. We report here a case of multifocal biliary adenoma affecting both the gallbladder and distal common bile duct in a patient who presented with progressive obstructive jaundice.

## Case presentation

A 70-year-old man with a past medical history of hypertension and diabetes mellitus presented to the hospital with a six-month history of progressive right upper quadrant abdominal pain and painless jaundice. Examination revealed scleral icterus and a non-tender palpable mass in the right upper quadrant. Laboratory tests showed elevated total bilirubin (17.8 mg/dL), direct bilirubin (10 mg/dL), alkaline phosphatase (1,316 IU/L), alanine aminotransferase (75 IU/L), aspartate aminotransferase (73 IU/L), and carbohydrate antigen 19-9 (326 U/mL); hepatitis serologies were negative (Table 1).

**Table 1:** Laboratory data at initial evaluation.

Lab Data	Patient Value	Reference Range
Total bilirubin	17.8 mg/dL	0.2-1.2 mg/dL
Direct bilirubin	10.0 mg/dL	0.0-0.3 mg/dL
Alkaline phosphatase	1316 IU/L	44-147 IU/L
Alanine aminotransferase	75 IU/L	7-56 IU/L
Aspartate aminotransferase	73 IU/L	10-40 IU/L
Carbohydrate antigen 19-9	326 U/mL	0-37 U/mL

**Figure 1:**
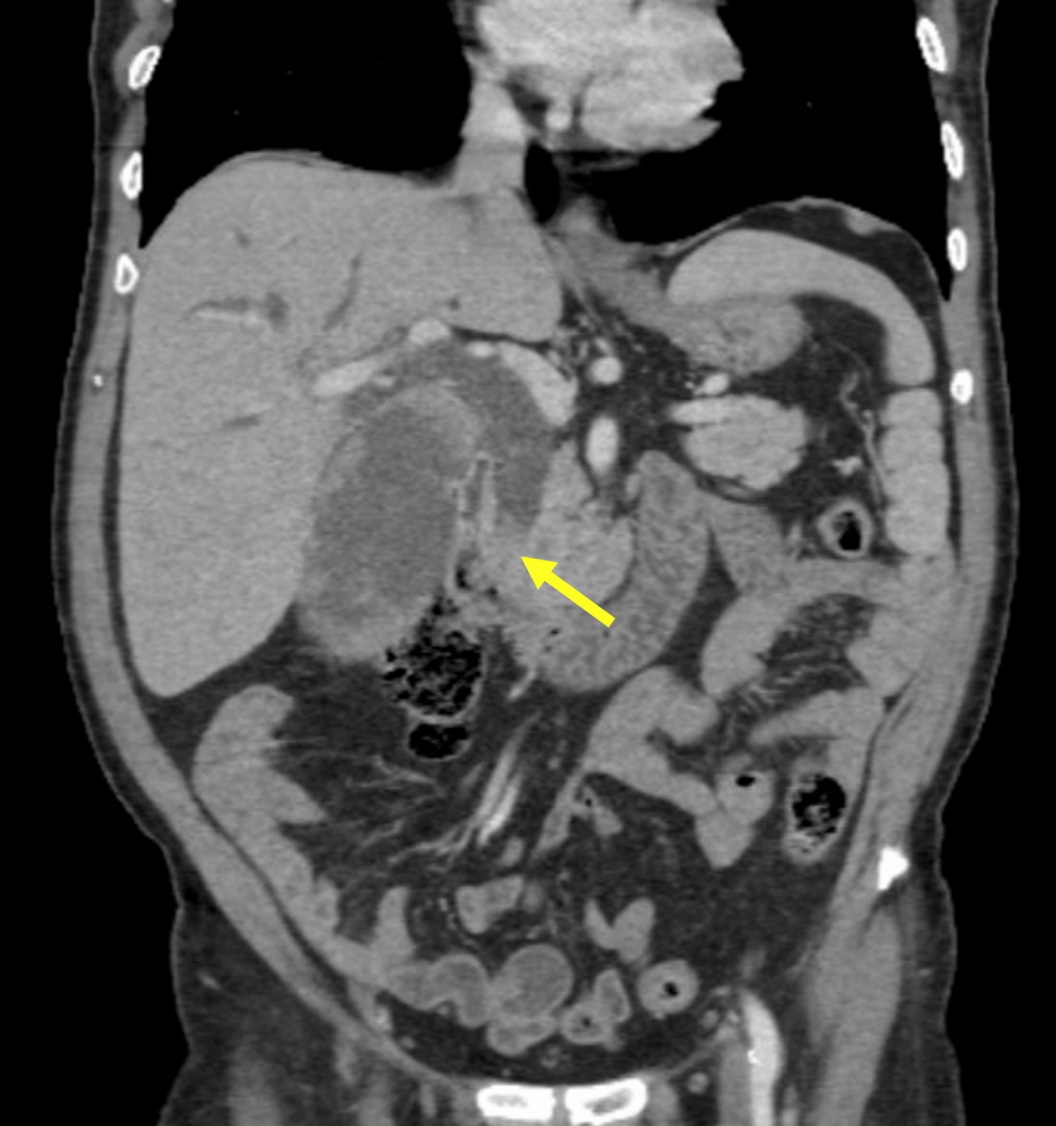
Coronal contrast-enhanced CT of the abdomen demonstrates diffuse dilation of the common bile duct with abnormal enhancement within the distal common bile duct (yellow arrow).

**Figure 2:**
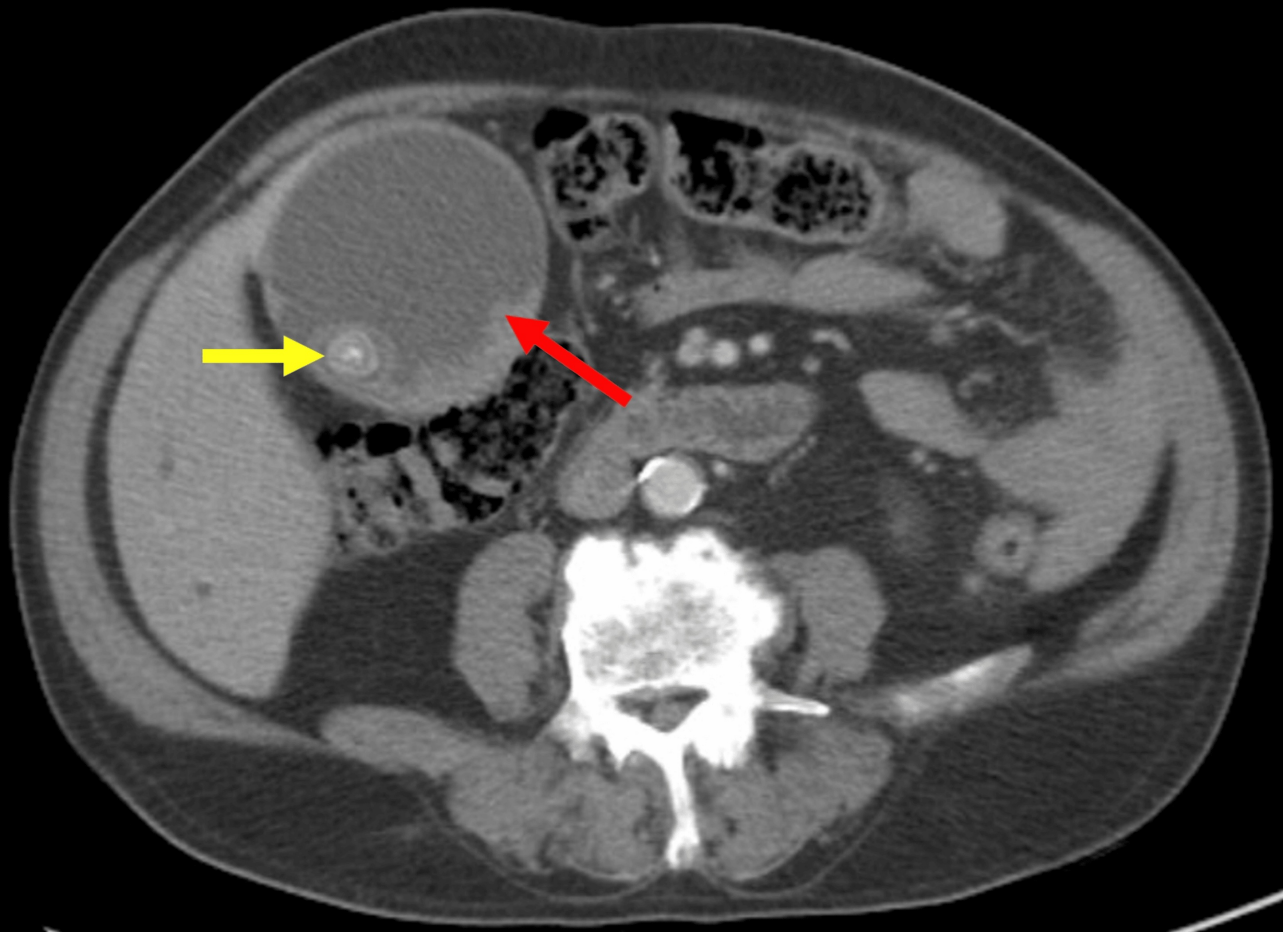
Axial contrast-enhanced CT of the abdomen shows markedly distended gallbladder with irregular wall thickening (red arrow) and intramural density (yellow arrow).

**Figure 3:**
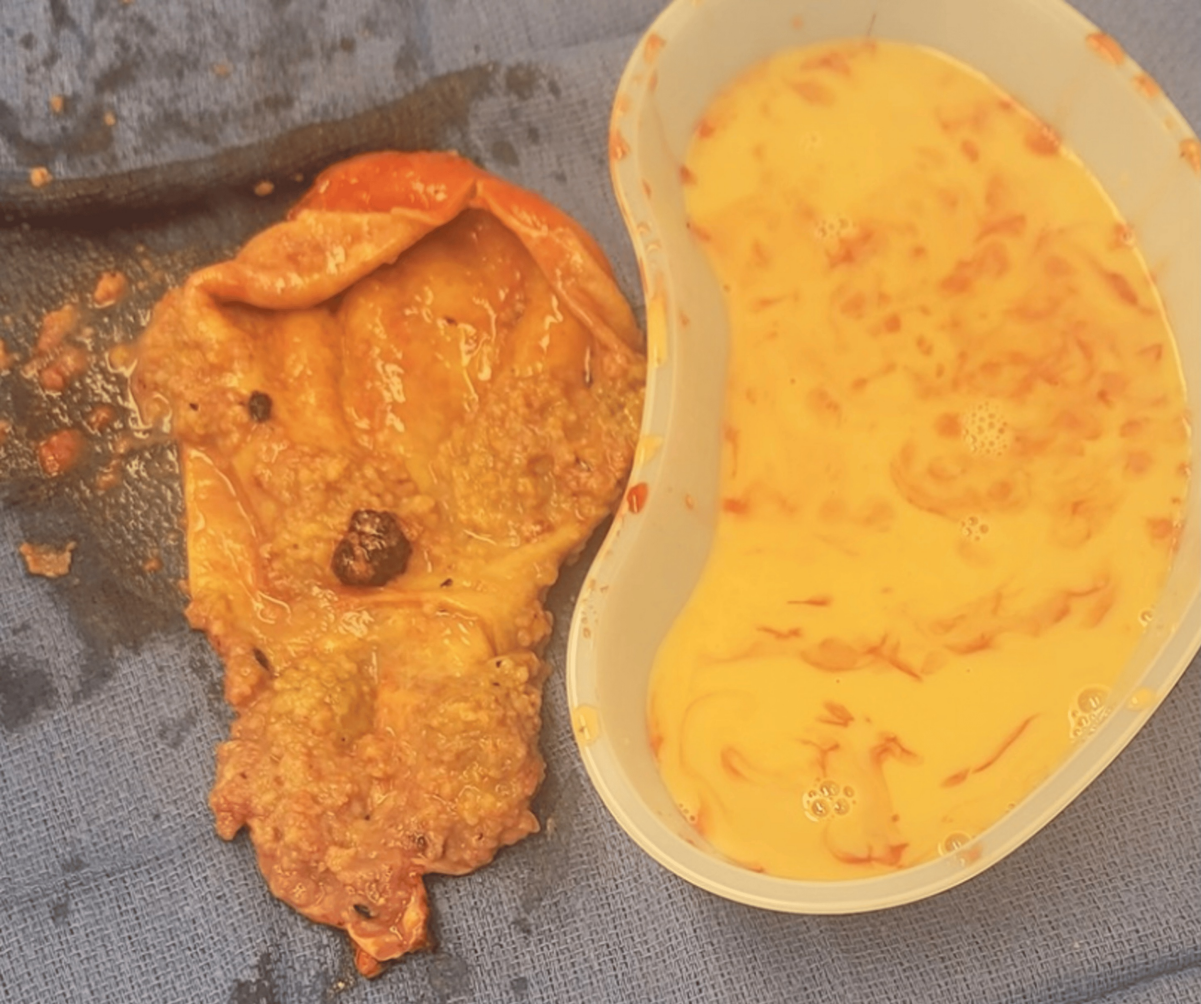
Gross intraoperative specimen of the gallbladder demonstrating diffuse thickening and shedding of polyploid tissue.

Abdominal computed tomography (CT) revealed a distended gallbladder with soft tissue enhancement along its wall and a dense lesion in the distal common bile duct (CBD), causing marked biliary dilation (Figures 1-2). Endoscopic retrograde cholangiopancreatography (ERCP) demonstrated a malignant-appearing stricture and a 15 mm mass in the distal CBD protruding into the duodenal lumen at the major papilla. A biopsy showed villous epithelium with adenomatous changes without high-grade dysplasia or malignancy. Despite these benign biopsy results, a strong clinical and radiologic suspicion for malignancy remained. Due to the suspicion of malignancy and the nature of the distal CBD tumor, a pancreaticoduodenectomy was initially planned for definitive diagnosis and treatment. However, intraoperative findings revealed a gallbladder polyp with diffuse polypoid tissue shedding into the CBD (Figure 3), prompting a transition to an exploratory laparotomy with open cholecystectomy. Frozen section histopathology of the gallbladder confirmed a tubulovillous adenoma without dysplasia or malignancy. This was consistent with tissue discovered during ERCP, suggesting that the obstruction in the distal CBD was caused by tissue shedding.

**Figure 4:**
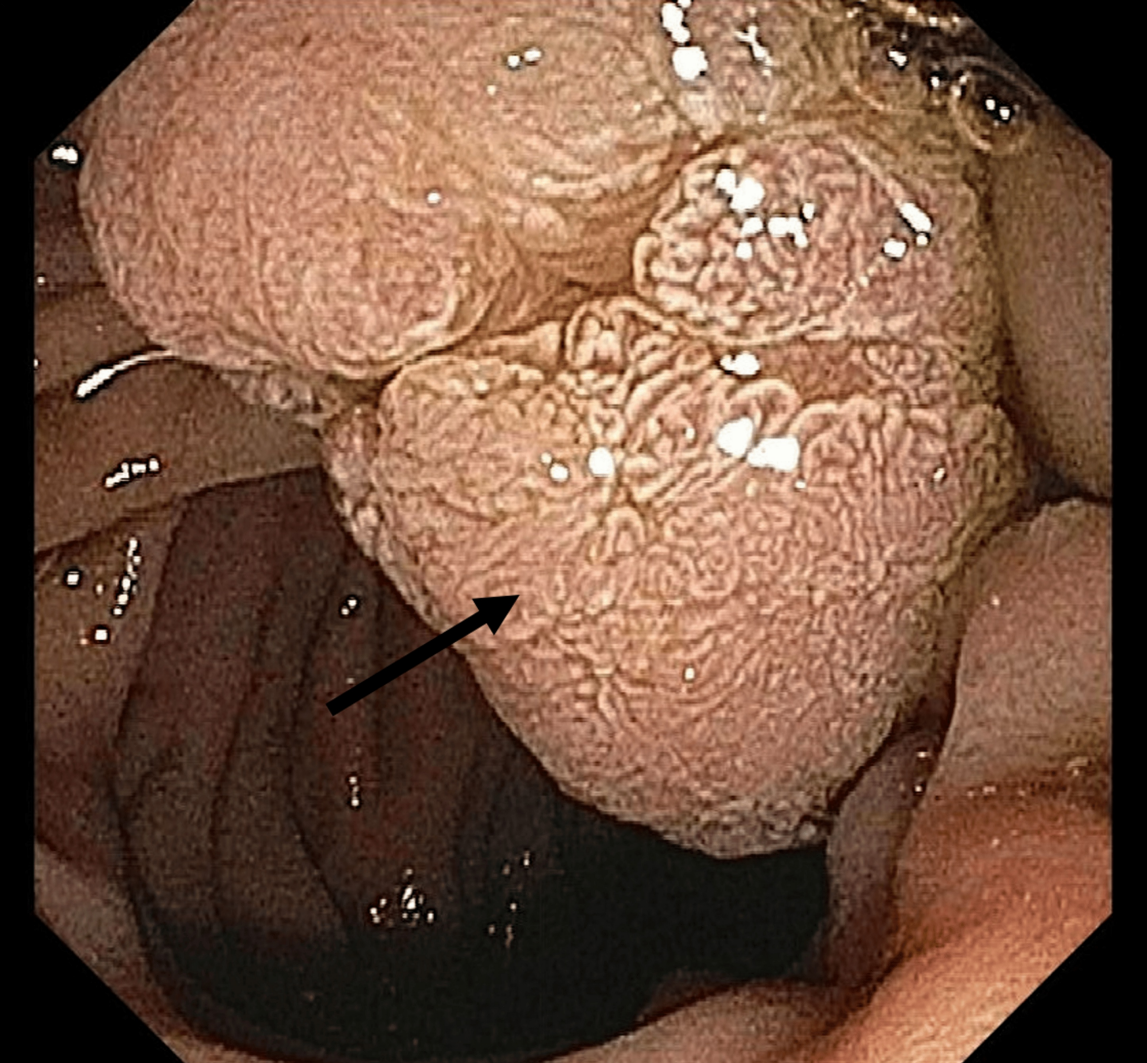
ERCP revealing a villous mass (black arrow) originating from the distal common bile duct protruding through the major papilla into the duodenal lumen. ERCP: Endoscopic retrograde cholangiopancreatography

**Figure 5:**
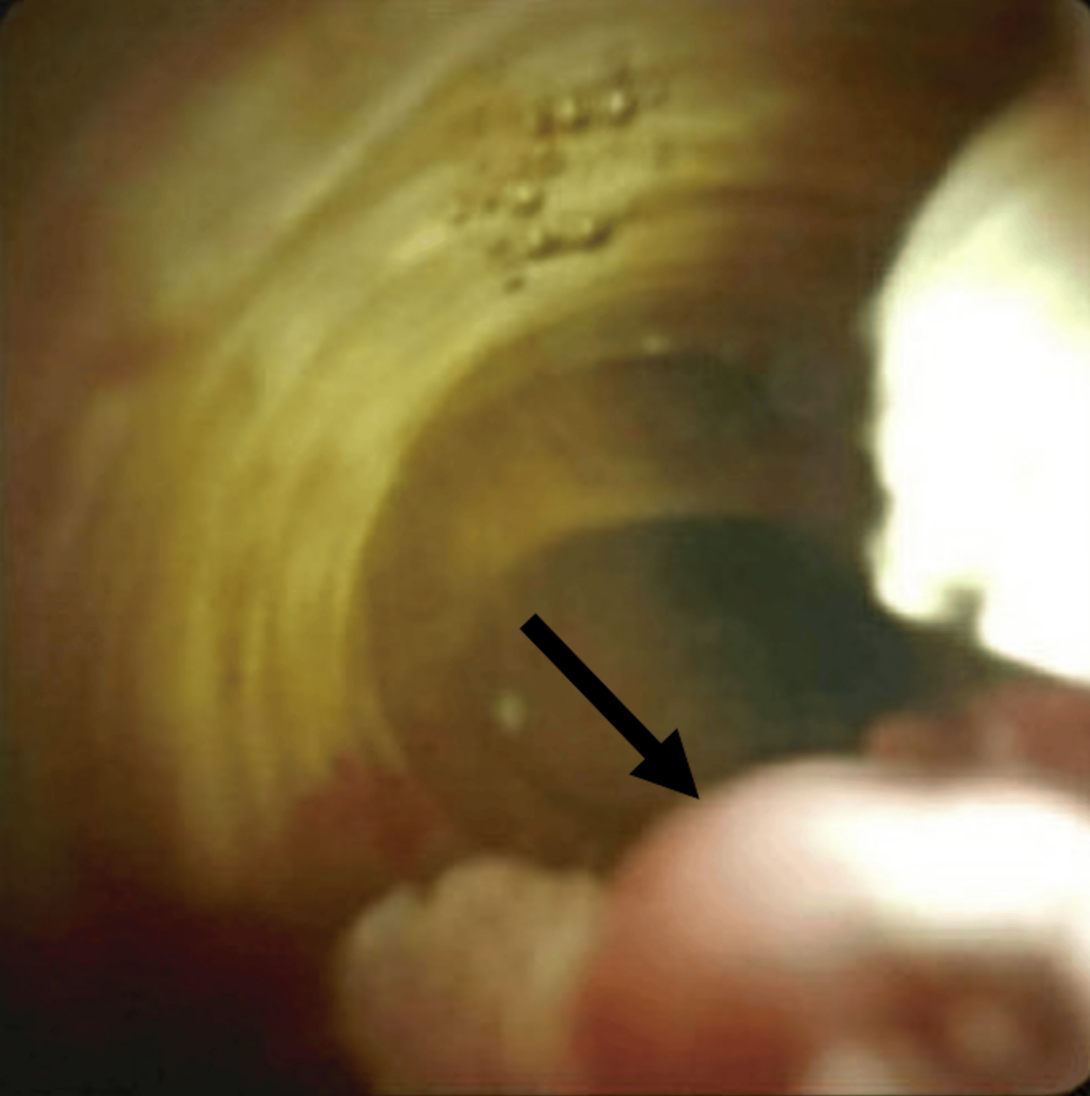
ERCP with cholangioscopy demonstrating an intraductal mass within the distal common bile duct (black arrow). ERCP: Endoscopic retrograde cholangiopancreatography

**Figure 6:**
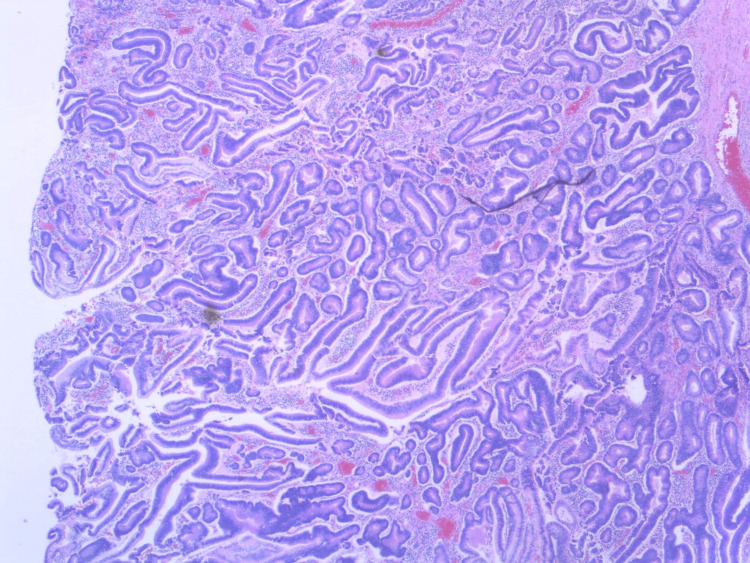
Low-power histologic section of the surgical specimen demonstrates a tubulovillous adenoma with features of low-grade dysplasia and no evidence of malignancy.

To further assess for additional intraductal neoplasms, repeat ERCP with endoscopic ultrasound (EUS) was performed. This demonstrated intraluminal growth of the ampullary lesion seen on initial ERCP into the distal CBD (Figures 4-5). Intraductal biopsies again showed tubulovillous adenoma without malignancy. The patient subsequently underwent a curative pancreaticoduodenectomy with pancreaticojejunostomy, hepaticojejunostomy, and gastrojejunostomy reconstruction. A final histopathology of the surgical specimen demonstrated a tubulovillous adenoma without evidence of high-grade dysplasia or malignancy (Figure 6). His postoperative course was uncomplicated, and at a three-month follow-up visit, he demonstrated resolution of jaundice and normalization of liver enzymes, with no evidence of tumor recurrence.

## Discussion

Benign tumors of the extrahepatic biliary system are rare and represent only a small subset of all extrahepatic bile duct masses [1, 3]. According to the World Health Organization, benign epithelial gallbladder and extrahepatic bile duct tumors are classified as adenoma, biliary intraepithelial neoplasia, intracystic papillary neoplasm, and intraductal papillary neoplasm of the bile duct [9]. Biliary adenomas are far more common in the gallbladder than in the common bile duct [2, 5]. These lesions may be classified as having a papillary/tubulopapillary morphology and resemble intestinal villous/tubulovillous adenomas [2]. Although benign, some biliary adenomas may progress to malignant lesions via the adenoma-to-carcinoma sequence [3, 4], making complete resection essential to prevent the development of carcinoma.

Gallbladder and extrahepatic biliary adenomas most often affect males in their sixth decade of life [2, 4, 5]. Most lesions remain asymptomatic until they grow large enough to cause complete obstruction of the bile duct. When this occurs, patients most commonly present with abdominal pain, obstructive jaundice, and complications such as cholangitis [1, 2, 5, 6]. Obstructive jaundice caused by biliary adenomas is clinically indistinguishable from that caused by malignancy. Notably, malignancy accounts for the majority of obstructive jaundice cases (59.4%), while benign etiologies are responsible for only 41.6% of cases [7]. Additionally, serum CA 19-9 has traditionally been used as a tumor marker for pancreatico-biliary malignancies. However, its diagnostic utility may be limited, as levels may rise in benign conditions, causing obstructive jaundice [10].

Radiographic imaging with abdominal CT and ultrasonography may reveal dilation of the biliary ducts, soft tissue enhancement, or wall thickening [1, 11, 12]. However, these imaging findings are non-specific and could mimic carcinoma and choledocholithiasis [1]. Endoscopic techniques that may be used to aid in the diagnosis of bile duct tumors include ERCP and EUS. The combined use of EUS with ERCP assists in the differentiation between benign and malignant biliary neoplasms, demonstrating an 88% diagnostic accuracy in distinguishing benign versus malignant strictures [13]. Tissue collection during endoscopic procedures is the only way to establish a definitive diagnosis. Despite the diagnostic utility of ERCP with EUS, endoscopic biopsies and brush cytology of biliary strictures and masses provide a definitive diagnosis of malignancy in only 36-46% of cases [14]. Therefore, definitive diagnosis of malignancy can only be made via postoperative histopathology [2, 5].

Management of extrahepatic biliary adenomas is not clearly defined. Endoscopic resection of intraductal adenomas with snare polypectomy or forceps has been successfully performed in a minority of cases; however, the risk of tumor recurrence is high [1, 3, 6, 8, 15]. Surgical resection is the mainstay of treatment. Local resection may be feasible for patients with benign tumors that demonstrate no signs of atypia or dysplasia [3-5]. Radical resection is recommended for any tumor with malignant features or those that are > 2 cm in size [4]. Pancreaticoduodenectomy should be considered mandatory for any type of biliary cancer involving the distal CBD, or if surgical margins are affected following initial resection [1, 3-5, 16]. The prognosis of extrahepatic biliary adenomas is typically favorable. However, villous/tubulovillous adenomas of the CBD may pose a malignancy risk, particularly in association with hereditary syndromes such as familial adenomatous polyposis or Gardner’s syndrome [2-4].

## Conclusions

In conclusion, adenomas are benign tumors of the extrahepatic biliary tree. While rare, their potential for malignant transformation necessitates a high index of suspicion and a thorough diagnostic workup. This case demonstrates multifocal biliary adenoma presenting with jaundice without underlying malignancy and emphasizes the importance of combining radiologic, endoscopic, and surgical assessments to guide management. Surgical resection should be considered the mainstay of treatment, and histopathologic evaluation is the gold standard for diagnosis.
